# Supplementary material for: Comparing prioritization strategies for delivering indoor residual spray (IRS) implementation, using a network approach
Source: Malar J. 2020 Sep 4;19:326. doi: 10.1186/s12936-020-03398-z (PMC7650283; doi:10.1186/s12936-020-03398-z)
Supplement: Supplementary file 1 — Additional file 1: Table S1. Number of city centre points and communities, and number included in network analyses, for the four study provinces in Zambia. Table S2. Kappa statistics for prioritizing communities (N = 3218) between pairs of prioritization strategies, within each district. Table S3. Number of communities to receive IRS, as prioritized under the four strategies (PfPR, An. arabiensis, An. funestus, An. gambiae) when spraying half of all households across all four provinces. Table S4. Kappa statistic for prioritizing communities (N = 3218) between pairs of prioritization strategies, when spraying half of all houses across all four provinces. [file 12936_2020_3398_MOESM1_ESM.docx]

**Additional File**

**Table S1.** Number of city centre points and communities, and number included in network analyses, for the four study provinces in Zambia

|  | Luapula | Muchinga | Northern | Eastern | Total |
| --- | --- | --- | --- | --- | --- |
| City centre points | 50 | 66 | 59 | 61 | 236 |
| *City centre points captured* | *50* | *61* | *58* | *59* | *228* |
| Total Communities | 758 | 5088 | 8241 | 4350 | 18,437 |
| *Communities captured* | *693* | *3140* | *5050* | *2363* | *11,146* |

**Table S2.** Kappa statistics for prioritizing communities (N=3,218) between pairs of prioritization strategies, *within each* district.

| Strategy 1 | Strategy 2 | Kappa statistic | *P*-value^1^ |
| --- | --- | --- | --- |
| *Pf*PR | *An. arabiensis* | -0.1160 | 1.000 |
| *Pf*PR | *An. funestus* | 0.1023 | < 0.001 |
| *Pf*PR | *An. gambiae* | 0.1192 | 1.000 |
| *An. arabiensis* | *An. funestus* | -0.0055 | 0.6621 |
| *An. arabiensis* | *An. gambiae* | -0.1366 | 1.000 |
| *An. funestus* | *An. gambiae* | -0.1935 | 1.000 |

^1^for agreement

**Table S3.** Number of communities to receive IRS, as prioritized under the four strategies (*Pf*PR, *An. arabiensis,* *An. funestus, An. gambiae*) when spraying half of all households *across all four provinces.*

|  | Number of communities | Percent of communities |
| --- | --- | --- |
| Prioritized by zero strategies | 148 | 3.1% |
| Prioritized by one strategy | 1,186 | 24.9% |
| Prioritized by two strategies | 2,296 | 48.2% |
| Prioritized by three strategies | 1,012 | 21.2% |
| Prioritized by four strategies | 126 | 2.6% |

**Table S4.** Kappa statistic for prioritizing communities (N=3,218) between pairs of prioritization strategies, when spraying half of all houses *across all four provinces*.

| Strategy 1 | Strategy 2 | Kappa statistic | *P*-value^1^ |
| --- | --- | --- | --- |
| *Pf*PR | *An. arabiensis* | -0.2483 | 1.000 |
| *Pf*PR | *An. funestus* | 0.0261 | 0.0359 |
| *Pf*PR | *An. gambiae* | 0.1127 | < 0.001 |
| *An. arabiensis* | *An. funestus* | 0.2332 | < 0.001 |
| *An. arabiensis* | *An. gambiae* | -0.2812 | 1.000 |
| *An. funestus* | *An. gambiae* | -0.4595 | 1.000 |

^1^for agreement
